# Overexpression of CUGBP1 in Skeletal Muscle from Adult Classic Myotonic Dystrophy Type 1 but Not from Myotonic Dystrophy Type 2

**DOI:** 10.1371/journal.pone.0083777

**Published:** 2013-12-20

**Authors:** Rosanna Cardani, Enrico Bugiardini, Laura V. Renna, Giulia Rossi, Graziano Colombo, Rea Valaperta, Giuseppe Novelli, Annalisa Botta, Giovanni Meola

**Affiliations:** 1 Laboratory of Muscle Histopathology and Molecular Biology, IRCCS-Policlinico San Donato, Milan, Italy; 2 Department of Neurology, University of Milan, IRCCS-Policlinico San Donato, Milan, Italy; 3 Department of Biosciences, University of Milan, Milan, Italy; 4 Department of Biomedicine and Prevention, Tor Vergata University of Rome, Rome, Italy; 5 Research Laboratories - Molecular Biology, IRCCS-Policlinico San Donato, Milan, Italy; 6 IRCCS-Neuromed, Pozzilli, Isernia, Italy; Colorado State University, United States of America

## Abstract

Myotonic dystrophy type 1 (DM1) and type 2 (DM2) are progressive multisystemic disorders caused by similar mutations at two different genetic loci. The common key feature of DM pathogenesis is nuclear accumulation of mutant RNA which causes aberrant alternative splicing of specific pre-mRNAs by altering the functions of two RNA binding proteins, MBNL1 and CUGBP1. However, DM1 and DM2 show disease-specific features that make them clearly separate diseases suggesting that other cellular and molecular pathways may be involved. In this study we have analysed the histopathological, and biomolecular features of skeletal muscle biopsies from DM1 and DM2 patients in relation to presenting phenotypes to better define the molecular pathogenesis. Particularly, the expression of CUGBP1 protein has been examined to clarify if this factor may act as modifier of disease-specific manifestations in DM. The results indicate that the splicing and muscle pathological alterations observed are related to the clinical phenotype both in DM1 and in DM2 and that CUGBP1 seems to play a role in classic DM1 but not in DM2. In conclusion, our results indicate that multisystemic disease spectrum of DM pathologies may not be explained only by spliceopathy thus confirming that the molecular pathomechanism of DM is more complex than that actually suggested.

## Introduction

Myotonic dystrophy (DM) is the most common adult onset muscular dystrophy affecting mainly skeletal muscle, heart, and the central nervous system [1]. Two DM loci are associated with two types of the disease. DM type 1 (DM1) is caused by the expansion of an unstable CTG trinucleotide repeat in the 3′ untranslated region of the DM protein kinase (*DMPK*) gene [2], [3]. The DM type 2 (DM2) mutation consists in the expansion of an unstable CCTG tetranucleotide within the first intron of the CCHC-type zinc finger, nucleic acid-binding protein (*CNBP*) gene (previously named *Zinc Finger Protein 9, ZNF9* gene) [4]. Both DM1 and DM2 are progressive multisystemic disorders characterized by muscle weakness, myotonia, cataracts, cardiac conduction defects, cerebral involvement and endocrinological disturbances such as increased insulin resistance and male hypogonadism. Experimental evidence supports an RNA gain-of-function mechanism of expanded transcripts in both DM1 and DM2 in which repeat containing transcripts from the expanded allele accumulate in nuclei as foci and alter the functions of RNA binding proteins involved in regulating alternative splicing and mRNA translation [5], [6]. The alteration of pre-mRNA processing strengthens the hypothesis of a spliceopathy which leads to inappropriate expression of embryonic splicing isoforms in adult tissues thus explaining, at least in part, the multisystemic aspect of the disease [7]. Expanded CUG/CCUG repeats mediate their effects on alternative splicing regulation through at least two RNA binding proteins: muscleblind like 1 (MBNL1) and CUGBP/Elav-like family member 1 (CELF1/CUGBP1) [8]. MBNL1 preferentially recognizes CUG or CCUG repeats when they are pathologically expanded [9], [10] and is sequestered by ribonuclear foci in DM1 and DM2 cells [9], [11], [12] resulting in a loss of MBNL1 activity. In contrast, CUGBP1 does not colocalize with ribonuclear foci in DM1 cells [9], [13], [14], however this protein may have a role in the pathogenesis of splicing abnormalities because it is overexpressed in DM1 myoblasts, skeletal muscle and heart tissues [15]–[17].

Although DM1 and DM2 have similar clinical and genetic characteristics, they also present a number of very dissimilar features. DM1 is characterized by the phenomenon of anticipation, by which the disease has an earlier onset and more severe course in subsequent generations. Thus the clinical spectrum of DM1 include four main categories, each presenting specific clinical features: the congenital form that presents the most severe phenotype characterized mainly by CNS involvement and mental retardation, the childhood-onset form with school mating and psychological problems, the adult-onset (“classical” DM1) where the core features are facial weakness with ptosis, myotonia and distal muscle weakness, and the late-onset or oligosymptomatic patients where only limited features are found on clinical and paraclinical assessment. The DM1 mutation length predicts the clinical outcome to some extent: oligosymptomatic 50–100 repeats, classical DM1 100–1.000 repeats; congenital >1.000 repeats [18], [19]. There is a relative correlation between the length of CTG repeat expansions and age of onset for DM1 patients with CTG <400, but correlation between repeat length and disease severity is poor for long repeats [1], [20], [21]. In DM2 there are no distinct clinical subgroups although initially, different phenotypes of DM2 with proximal muscle weakness were described: DM2/Proximal Myotonic Myopathy (PROMM) and Proximal Myotonic Dystrophy (PDM) [22]–[25]. PDM patients show many features similar to those found in PROMM, including proximal muscle weakness, cataracts, and electrophysiologically detectable myotonia. Unlike PROMM patients, however, they do not report myalgias, symptomatic myotonia, or muscle stiffness. Instead they present traits not present in PROMM, such as pronounced dystrophic-atrophic changes in the proximal muscles and late-onset progressive deafness [24]. The most important discrepancy between DM1 and DM2 is absence of a congenital form in DM2 [26], [27]. In DM2 the smallest reported mutation vary between 55–75 CCTG [4], [28] and the largest expansions have been measured to be up about 11.000 repeats [4], however the size of CCTG repeat expansion in leukocyte DNA in DM2 seems to relate in large part to the age of the patient and not necessarily to the severity of symptoms or manifestations. Despite the CCUG expansions are longer than DM1 CUG expansions, DM2 shows a less severe phenotype. Clinical myotonia is usually milder in DM2 and histopathological features in DM1 and DM2 are also different. In DM2 a subpopulation of extremely atrophic type 2 fibers, including the nuclear clump fibers, are present [1], [29], [30].

Recent studies have indicated that cardinal features of DM1 can be reproduced in the absence of nuclear inclusions and that RNA foci formation and splicing defects are separable [31], [32]. Moreover, DM1-associated splicing defects have been observed in mouse models of other muscular dystrophies indicating that spliceopathy is secondary to muscle damage [33]. However to date, literature has been focused on reinforcing the prevailing common model of DM pathogenesis based on the presence of mutant RNA foci in cell nuclei and spliceopathy. On the other hand, the existence of disease-specific features that make DM1 and DM2 clearly separate diseases and the existence of DM1 and DM2 distinct subtypes suggest that other cellular and molecular pathways are involved besides the shared pathogenetic model hypothesized. Moreover, the RNA gain of function toxicity has been better characterized in DM1 than in DM2 probably due to a greater availability of DM1 samples and mouse models. Importantly, the role of CUGBP1 in DM2 is particularly intriguing with contradictory results being reported. Indeed it appears that in DM1 a combined effect of decreased MBNL1 and increased CUGBP1 activity lead to misregulated alternative splicing and other changes of the muscle transcriptome [5], [34]. Instead in DM2, splicing abnormalities are also associated with the sequestration of MBNL1 protein by expanded transcripts [5], [12], however evidence that CUGBP1 upregulation also occurs in DM2 is conflicting [34]–[36]. Timchenko and colleagues reported an increase of CUGBP1 in DM2 cultured myoblasts and muscle biopsies analyzing cytoplasmic extracts [34]. Moreover they reported that expression of pure RNA CCUG repeats in normal human myoblasts, in C2C12 cells and in a DM2 mouse model also increased levels of CUGBP1 [34]. On the contrary, in two different reports, the analysis of total cellular extract from DM2 cultured myoblasts and from muscle biopsies of DM2 patients did not show differences in CUGBP1 levels [35], [36]. Nevertheless, it should be noted that in these works no mention is made of either the number or the clinical features and muscle histopathology of the patients used. In DM2 patients the role of ZNF9/CNBP expression is still controversial and requires additional investigation since some DM2 patients show reduced protein levels but others do not [36], [37]–[40]. In this study we have analysed the histopathological, biochemical and molecular features of skeletal muscle biopsies from DM1 and DM2 patients in relation to presenting phenotypes (mild-E1 *vs.* classic-E2 *vs.* CDM in DM1 and PROMM *vs.* PDM *vs.* paucisymptomatic in DM2). This is the first study where the expression of CUGBP1 protein has been examined in a large cohort of DM2 patients. Moreover, DM2 muscle biopsies have been characterized together with several DM1 and control samples. This work intends to clarify which factors may act as modifiers of disease-specific manifestations in DM beyond spliceopathy.

## Materials and Methods

### Patients and skeletal muscle samples

This study was authorized by the Institutional Ethics Committee (ASL MI2-Melegnano via VIII Giugno, Milan) and was conducted according to the principles expressed in the Declaration of Helsinki, the institutional regulation and Italian laws and guidelines. All blood samples and muscle biopsies were used for this study after receiving written informed consent from the patients. With regard to children participants, we have obtained written informed consent from their parents.

Human muscle biopsies from biceps brachii muscle were taken under sterile conditions from 18 DM1, 20 DM2 patients and from 8 age-matched subjects who underwent muscle biopsy and resulted negative. Muscle samples were trimmed of blood vessels, fat and connective tissues and then fresh-frozen in isopentane cooled in liquid nitrogen. The diagnosis of DM was based upon the clinical diagnostic criteria set by the International Consortium for Myotonic Dystrophy [41]. Fluorescence in situ hybridization was performed on DM2 muscle frozen sections using a (CAGG)_5_ probe as previously reported by Cardani et al. [42] to verify the presence of ribonuclear inclusions.

### Genetic analysis of CTG and CCTG expansions

For DM1 genotyping, 1 µg of genomic DNA of each patients extracted from peripheral blood leukocytes were by “*Myotonic Dystrophy SB kit*” (Experteam s.r.l, Venezia, Italy). Forward primer was labelled at the 5’ end with fluorescent tag 6-FAM. PCR conditions were: one cycle of 1 min at 94°C; 28 cycles of 20 sec at 94°C and 7 min at 62°C; and finally 10 min at 72°C. The amplifications were performed by MyCycler instrument (BioRad). After the amplification 20 µl of each PCR products were run on 3.5% MetaPhore agarose gel at 100V and stained with ethidium bromide. Alleles with less than 100 repeats were analyzed by capillary electrophoresis on 3500 Genetic Analyzer (Applied Biosystems) using LIZ600 as size standard. The analysis of results was performed using GeneMapper v4.1 (Applied Biosystems). For alleles with more than 100 repeats Southern blot hybridization was performed using a non-radioactive Digoxigenin-based probe 5’DIG- labelled [CTG]_10,_ and the [CTG] repeats size was determined comparing the bands pattern obtained by Southern Blot Analysis with two DNA Molecular Weight Markers VII and VIII, DIG-labelled (Roche Diagnostics). DM2 genotyping has been performed on genomic DNA extracted from peripheral blood leukocytes by long-PCR analysis as described [37], [43].

### Muscle histopathology

Muscle tissue was fresh-frozen in isopentane cooled in liquid nitrogen. Histopathological analysis was performed on serial sections (8 µm) processed for routine histological or histochemical stainings. A standard myofibrillar ATPase staining protocol was used after preincubation at pH 4.3, 4.6, and 10.4 [44]. The most typical alterations, such as nuclear clump fibers (i.e. aggregates of myonuclei with a thin rim of cytoplasm), nuclear centralization and fiber size variability were evaluated on serial muscle sections.

### Immunohistochemistry

Serial transverse muscle cryostat sections 6 µm thick were cut for immunohistochemical staining (IHC). Sections were air-dried and rehydrated in phosphate buffer pH 7.4 (PBS). Non-specific binding sites were blocked with normal goat serum (NGS; DAKO) at a dilution 1∶20 in PBS containing 2% bovine serum albumin (BSA; Sigma-Aldrich) for 20 min at room temperature (RT). Mouse monoclonal primary antibodies against two different myosin heavy chain (MHC) isotypes were used at the following dilutions: MHCfast, 1∶400 in PBS+2% BSA (Sigma-Aldrich); MHCslow, 1∶400 in PBS+2% BSA (Sigma-Aldrich). Each antibody was applied for 1 h at RT. After washing in PBS 3 times for 5 min, sections were incubated with goat anti-mouse biotinylated secondary antibody diluted 1∶300 in PBS+2% BSA for 1 h at RT. After PBS washing, sections were incubated with StreptABComplex (DAKO) for 30 min and then exposed to the 3,3’-diaminobenzidine tetrahydrochloride (DAB) chromogen reaction solution for 10 min. Nuclei were counterstained with Mayer’s hematoxylin. Quantitative evaluation of fiber diameter was made as described previously by Vihola et al. [29] on images taken with a slide scanner ScanScope CS (Aperio Technologies, Vista, CA, USA) using the slide scanner software ImageScope. The size of muscle fibers was assessed by measuring the ‘‘smallest fiber diameter.’’ All data were elaborated using Microcal Origin (Microcal Software Inc., Northampton, MA, USA).

### Western blot analysis

Whole cell extracts were obtained from fifteen-twenty consecutive muscle cryostat sections 10 µm thick homogenized in 60 µl of 50 mM TrisHCl with 5% SDS (pH 7.5). After incubating on ice for 15 min, samples were centrifuged at 5,700 *g* for 12 min at 4°C, and supernatant was collected and stored at −80°C. Pellets were resuspended in 50 mM TrisHCl with 5% SDS (pH 7.5) and stored at −80°C. Protein concentration in each sample was determined by using BCA Protein Assay (Bio-Rad Laboratories). An equal amount of protein was loaded per lane and electrophoresed on 12% sodium dodecyl sulfate–polyacrylamide gels, and then transferred to nitrocellulose Protran membranes (Schleicher & Shuell GmbH). After blocking non specific sites in TrisHCl buffer pH 7.5 (TBS) containing 5% BSA for 30 min at 42°C, membranes were incubated overnight at 4°C with rabbit polyclonal anti-CUGBP1-posphoS28 (Abnova; 0.5 µg/ml), with mouse monoclonal anti CUGBP1 (Santa Cruz; clone 3B; 1∶1000), or with rabbit polyclonal anti-ZNF9/CNBP (1∶1000) [45]. After several washes in TBS+0.2% Tween20 or TBS+0.3% Tween20, membranes were incubated with horseradish peroxidase-conjugated goat anti-mouse or anti-rabbit secondary antibodies (Jackson ImmunoResearch Laboratories) diluted 1∶5000 or 1∶10000 in TBS+5% BSA+ 0.2% Tween20 respectively. Membranes were washed and immune complexes were detected using the ECL detection system (Amersham Pharmacia Biotech, Piscataway, NJ). GAPDH (polyclonal antibody diluted 1:80000; Sigma–Aldrich) was used as internal control to verify and correct for loading error. Blots have been performed in triplicate.

### Two-dimensional gel electrophoresis (2D-GE)

6 DM1 (3 DM1-E1, 3 DM1-E2), 6 DM2 (3 DM2-PDM, 3 DM2-PROMM) and 6 control samples have been analysed. Each sample containing 50 µg proteins was resuspended in a solution containing 7 M urea, 2 M thiourea, and 4% 3-((3-cholamidopropyl)-dimethylammonio)-1-propanesulfonate (CHAPS). Samples were used to rehydrate immobilized pH gradient (IPG) strips just before isoelectrofocusing. For the first-dimension electrophoresis, samples were applied to IPG strips (11 cm, pH 3–10 linear gradient; GE Healthcare). Strips were rehydrated at 20°C for 1 h without current and for 12 h at 30 V in a buffer containing 7 M urea, 2 M thiourea, 4% CHAPS, 1 mM dithiothreitol (DTT), and 1% IPG buffer 3–10 (GE Healthcare). Strips were focused at 20°C for a total of 70,000 V/h at a maximum of 8000 V using the Ettan IPGphor II system (GE Healthcare). The focused IPG strips were stored at −80°C. For the second dimension, IPG strips were equilibrated at room temperature for 15 min in a solution containing 6 M urea, 2% SDS, 30% glycerol, 50 mM Tris–HCl (pH 8.8), and 10 mg/ml DTT and then reequilibrated for 15 min in the same buffer containing 25 mg/ml iodoacetamide in place of DTT. The IPG strips were placed on top of a 12% polyacrylamide gel and proteins were separated at 25°C with a prerun step at 20 mA/gel for 1 h and a run step at 30 W/gel for 3.5 h. After run, gels were transferred to nitrocellulose Protran membranes (Schleicher & Shuell GmbH). CUGBP1 and GAPDH, used to normalize protein load on IPG strip, have been immunodetected as described above.

### Study of alternative splicing

Frozen muscle samples were practiced for the extraction of total RNA using TRIzol reagent (Gibco BRL, Gaithersburg, MD) and 1 µg of RNA was reverse transcribed according to the cDNA protocol of the High Capacity cDNA Archive kit (Applied Biosystems, Foster City, CA). Splicing pattern profile of the *IR*, *CLCN1*, *MBNL1, SERCA1* and *CAPZB* genes was carried out as described [46]–[48]. Total PCR products, obtained within the linear range of amplification, were electrophoresed on 2.5% agarose gel. Quantitative analysis of the amplified products was performed using SybrGreenII-stained gels (Perkin-Elmer Life Science, Massachusetts, USA) scanned on a fluorimager 595 (Amersham Biosciences, Buckinghamshire, UK). The intensity of each band and the fraction of abnormally (or pathologically) spliced (AS) isoforms (AS-isoforms/total) were quantified by densitometry using ImageQuant software. Statistical methods were used to analyze the differences in the identified splice variants between DM1 and DM2 patients respect to controls. Control of the RT-PCR reaction was based on the expression level of the glucose phosphate isomerase housekeeping gene (GPI) and all amplifications have been carried out in triplicate using independent cDNA samples.

### QRT-PCR expression analysis of the CLCN1, ZNF9/CNBP and CUGBP1 genes

Following RNA extraction and retro-transcription, cDNA of DM samples were also used to quantify the expression level of the *CLCN1, ZNF9/CNBP* and *CUGBP1* genes. The total expression of mentioned genes was evaluated using specific TaqMan gene expression assays: *CLCN1* [Hs00163961_m1], *ZNF9/CNBP* [Hs00231535_m1] and *CUGBP1* [Hs00198069_m1] (Applied Biosystems). The VIC-labelled β_2_-microglobulin gene (*B2M*: GenBank accession #NM_004048) was used as housekeeping internal control gene, as described [49]. The simultaneous measurement of genes-FAM/B2M-VIC expression allows to normalize the amount of cDNA added per sample. Each PCR reaction was performed in triplicate using the TaqMan Universal PCR Master Mix and the ABI PRISM 7500 Fast System (Applied Biosystems). A comparative threshold cycle (Ct) was used to determine *CLCN1* and *ZNF9/CNBP* genes expression compared to a calibrator (median value of control subjects). Hence, steady-state mRNA levels were expressed a *n*-fold difference relative to the calibrator. For each sample, genes’ Ct value was normalized using the formula ΔCt  =  Ct genes – Ct B2M. To determine relative expression levels, the following formula was used: ΔΔCt  =  ΔCt sample − ΔCt calibrator. The value adopted to plot relative gene expression was calculated using the expression 2^−ΔΔCt^. The relative quantification of the *CUGBP1* mRNA steady-state level was calculated using the Pfaffl equation accordingly to Pfaffl et al [50].

### Statistical analysis

Overall statistical significance has been calculated by using the Kruskal-Wallis test (non parametric ANOVA) and significant differences between groups have been determined using the Dunn’s multiple comparisons post-test.

## Results

### Patients

On the basis of clinical phenotype, DM1 cohort has been divided in three subphenotypes: 5 DM1 patients with mild phenotype (E1), 10 DM1 patients with classic phenotype (E2) and 3 DM1 adult patients with Congenital Myotonic Dystrophy phenotype (CDM). The DM2 cohort included: 5 DM2 patients with a paucisymptomatic (PS) phenotype, 5 DM2 patients with Proximal Myotonic Dystrophy (PDM) phenotype (severe atrophy and myotonia only at EMG) and 10 DM2 patients with Proximal Myotonic Myopathy (PROMM) phenotype (proximal muscle weakness and myotonia). Data on DM patients used in this study are reported in Table 1.

**Table 1:** Clinical data on DM patients used in this study.

	Phenotype	Clinical features	Age at biopsy	MRC^a^	Diabetes	EKG^b^ Abnormalities %
				tot	%	
**Controls**	Healthy subjects	No clinical signs	45.2±13.8	149.3±1.0	0%	0%
**(n = 8)**						
**DM1 (n = 18)**	Mild (n = 5)	Minimal clinical signs	41.2±17.5	149.6±1.7	0%	0%
	(E1: 50<CTG<150)	(MIRS^c^ = 2)				
	Classic (n = 10)	Overt clinical symptoms	44.3±10.5	118.9±19.3	0%	60%
	(E2: 150<CTG<1000)	(MIRS = 3-4)				
	Congenital Myotonic Dystrophy (n = 3)	Symptoms at birth	22.7±10.7	114.7±4.2	0%	100%
	(CDM: CTG>1000)					
**DM2 (n = 20)**	Paucisymptomatic (n = 5)	Absence of muscular weakness	38.0±13.4	149.6±1.0	0%	0%
	(PS)					
	Proximal myotonic dystrophy (n = 5)	Severe atrophy	65.0±8.4	131.4±11.4	40%	0%
	(PDM)	No clinical myotonia				
	Proximal myotonic myopathy (n = 10)	Proximal muscle weakness	55.0±6.1	142.6±2.5	20%	20%
	(PROMM)	Myotonia				

a Medical Research Council, scale for muscle strength; scale (0–5 grade) on 15 muscles at both sides in the upper and lower limbs for a total of 150 maximum score.

b Electrocardiogram, included first-degree atrio-ventricular block, incomplete or complete bundle-branch block.

c Muscle Impairment Rating Scale, stage of the disease for DM1 patients [73].

### Muscle histopathology

**Figure 1:**
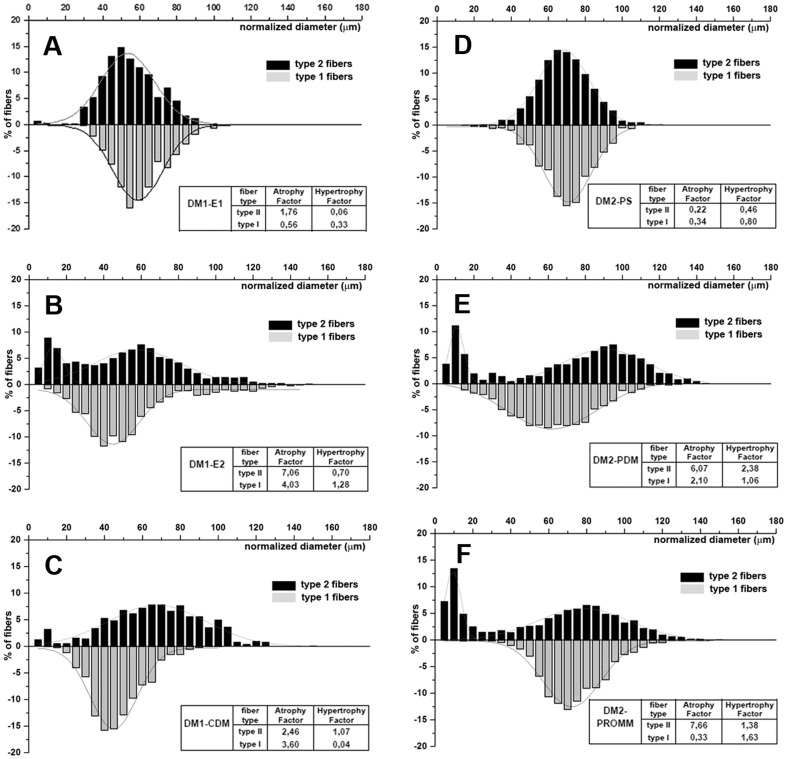
Metahistograms have been obtained from the analysis of muscle fiber diameters in DM1 patients (A-C) and in DM2 patients (D-F). The results are based on sections immunostained for MHC fast or slow myosin. Tables show the relative atrophy or hypertrophy factors in each subphenotype considered. Data relative to each DM1 and DM2 phenotypic groups have been obtained by pooling the findings of each patient: DM1-E1 (n = 3), DM1-E2 (n = 5), DM1-CDM (n = 3), DM2-PS (n = 4), DM2-PDM (n = 5) and DM2-PROMM (n = 5).

**Figure 2:**
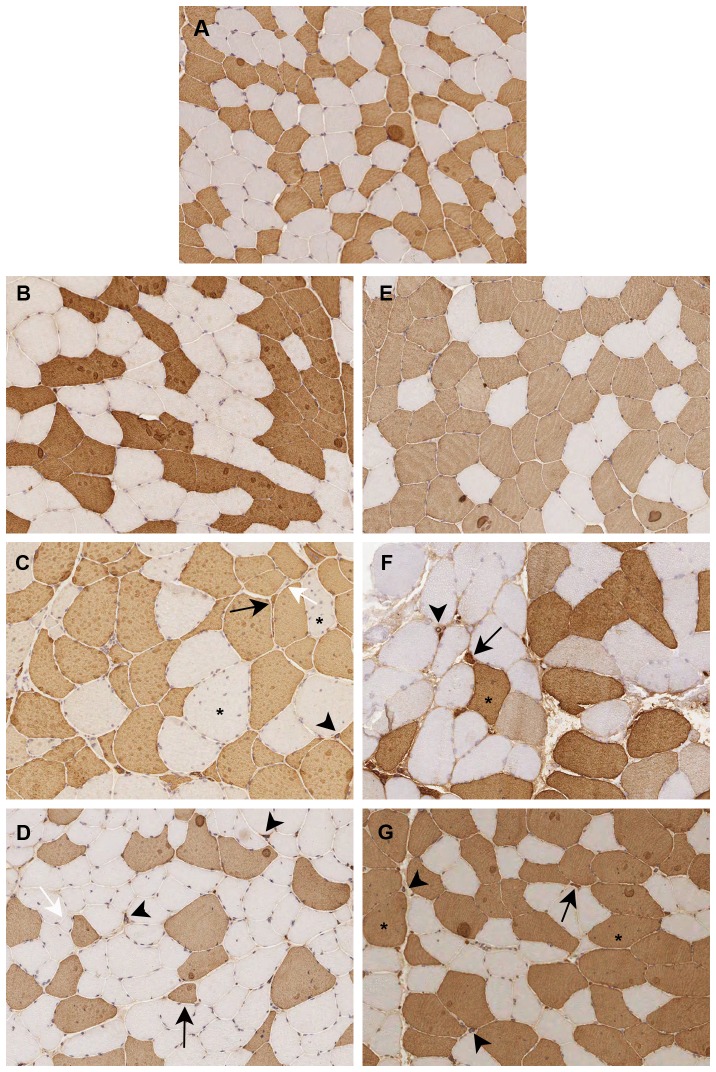
Fast myosin immunostaining of skeletal muscle transversal sections obtained from a healthy patient (A), DM1 patients (B-D) and DM2 patients (E-G). Type 2 fibers (fast positive fibers) are stained in brown. Muscle from DM1-E1 (B) and DM2-PS (E) patients show a normal histological muscle pattern similar to those observed in control muscle section (A). Muscle from DM1-E2 (C) and DM1-CDM (D) patients show a high fiber size variability with both type 1 (unstained fibers; white arrows) and type 2 (black arrows) atrophic fibers, fast positive nuclear clumps (arrowheads) and a preferential type 1 fiber central nucleation (asterisks). Muscle from DM2-PDM and DM2-PROMM patients also show high fiber size variability with very small type 2 fibers (black arrows), type 2 nuclear clumps (arrowheads) and a preferential type 2 fiber central nucleation (asterisks).

Analysis of muscle sections immunostained for MHC fast and slow myosin allows us to detect and measure fibers smaller than 5 µm, including all nuclear clump fibers which are recognizable by the presence of a thin rim of immunoreaction around the nuclei. The metahistograms based on the analysis of fiber diameters on immunostained muscle sections and the evaluation of atrophy (AF) and hypertrophy (HF) factors are reported in Figure 1. An increase of both type 1 and type 2 fiber AF is present in DM1-E2 and DM1–CDM (Figs. 1B, C). The AF increase is not present in DM1-E1 (Fig. 1A). Type 2 fiber atrophy is most evident in DM1-E2 which shows a bimodal size distribution histogram of type 2 fibers (Fig. 1B). Among DM2 muscles, DM2-PDM and DM2-PROMM show an increase of both AF and HF not evident in DM2-PS (Figs. 1D-F). In DM2-PROMM muscles, atrophy affects type 2 but not type 1 fibers whereas in DM2-PDM a slight increase of type 1 fiber AF is also present (Figs. 1E, F). Both DM2-PDM and DM2-PROMM exhibit a bimodal size distribution histogram of type 2 fibers. The most severe histopathological alterations are present in muscles from patients presenting the most severe clinical phenotype i.e. DM1-E2, DM1-CDM, DM2-PDM and DM2-PROMM. In DM1-E2 and DM1-CDM, nuclear clumps fibers and highly atrophic fibers express MHC fast myosin and a coexpression with MHC slow myosin is evident in most of them (Figs. 2C, D). In DM2-PDM and DM2-PROMM muscles, numerous nuclear clumps fibers expressing only MHC fast myosin are present (Figs. 2F, G). Central nucleation is always present and involved prevalently type 1 fibers in DM1 muscles and type 2 fibers in DM2 muscles. As in control muscles, no histopathological changes are instead observed in muscles from both DM1-E1 and DM2-PS where no nuclear clumps fibers or central nuclei are present (Figs. 2A, B, E).

### CUGBP1 protein expression is more elevated in DM1 than in DM2 skeletal muscle

**Figure 3:**
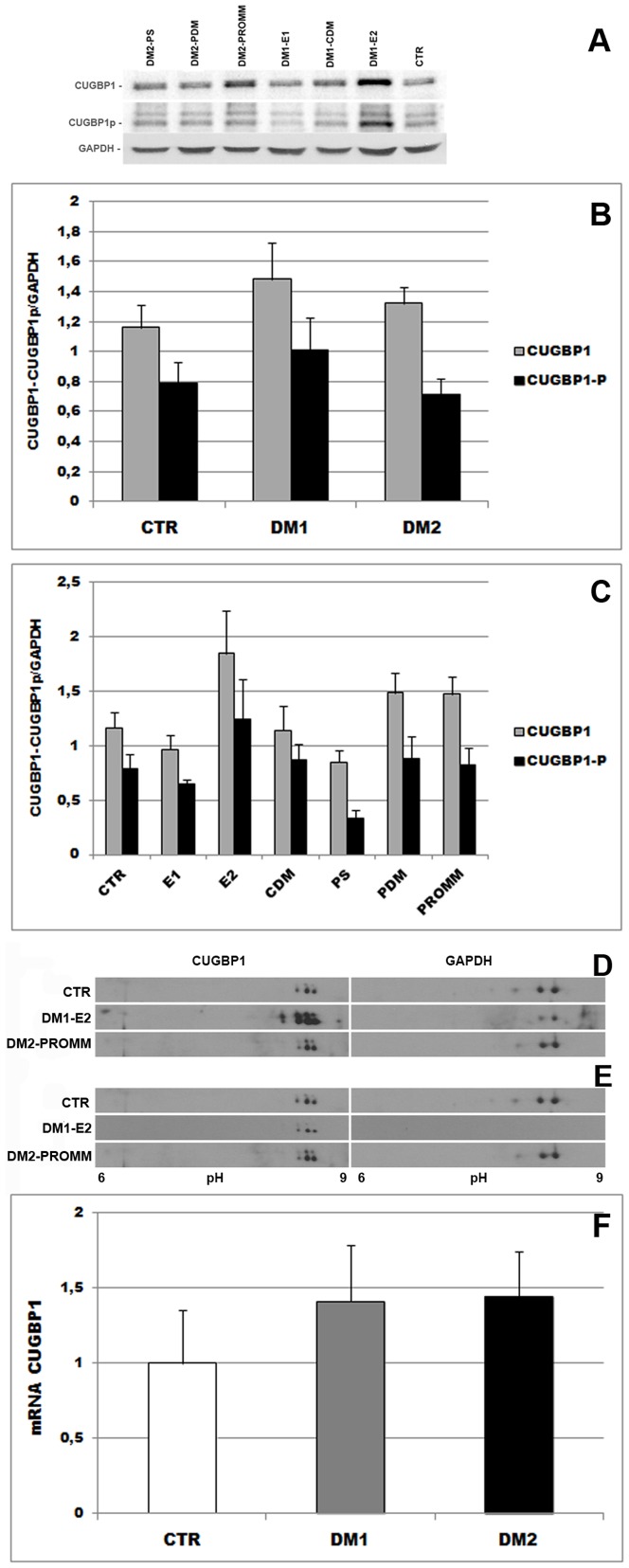
CUGBP1 expression in biceps brachii muscle samples from healthy, DM1 and DM2 patients. **A-E.** Analysis of CUGBP1 protein expression by western blot. **A**. Representative western blot analysis of CUGBP1 and CUGBP1-p-S28 protein expression in healthy, DM1 and DM2 patients. Density of the bands has been normalized with GAPDH expression used as internal control. **B.** Histograms represents mean values of CUGBP1 protein expression analysed by densitometry in DM1 (n = 18) and DM2 (n = 20) patients compared to controls (n = 8). Bars represent standard error of the mean (SEM). An increase of CUGBP1 and CUGBP1-p-S28 expression is more evident in DM1 muscles. **C.** CUGBP1 expression has been also evaluated in DM1 and DM2 phenotypic subgroups (DM1-E1 (n = 5), DM1-E2 (n = 10), DM1-CDM (n = 3), DM2-PS (n = 5), DM2-PDM (n = 5) and DM2-PROMM (n = 10)) compared to controls (CTR; n = 8). Bars represent SEM. Among all DM muscles analysed, the increase of CUGBP1 and CUGBP1-p-S28 levels is more evident in DM1-E2. **D.** Representative CUGBP1 protein expression pattern of 50 µg of biceps brachii muscle samples from healthy, DM1-E2 and DM2-PROMM patients determined by western blot analysis after 2D-GE separation (left panel). In order to evaluate protein load, GAPDH has been also detected in the 2D map (right panel). **E.** Since CUGBP1 is overexpressed in DM1-E2 (see Figure 3D), different exposition times (2 hours for DM2 and controls *vs* 5 minutes for DM1) have been compared to obtain similar western blot signals with the intent to compare the CUGBP1 phosphorylation patterns (protein distribution among the different CUGBP1 phosphorylated isoforms) in DM1, DM2 and control samples. As illustrated in the left panel, CUGBP1 phosphorylation pattern is not altered in DM1 and DM2 muscles as compared to controls. **F.** CUGBP1 mRNA expression in biceps brachii muscle samples from DM1 (n = 11), DM2 (n = 14) and controls (CTR, n = 4) patients. Bars represent standard deviation.

In order to resolve the controversial results on CUGBP1 protein expression in DM2 muscle, we have examined the protein levels of CUGBP1 in biceps brachii muscle samples from DM1, DM2 and control individuals by western blotting analysis (Fig. 3A). An increase of CUGBP1 protein level is present in DM muscles as compared to controls even if not statistically significant due to the high interindividual variability observed in all groups. However, the increase of protein expression appears to be higher in DM1 than in DM2 (Fig. 3B). When considering the 3 DM1 different phenotypes separately, the increase is evident only in DM1-E2 while in DM1-CDM and DM1-E1 muscles the protein levels appear to be equal to those observed in control muscles (Figs. 3C). A clear correlation between the AFs and the CUGBP1 expression levels has been observed in DM1 muscles (p<0.01, data not shown). A slight increase in CUGBP1 protein expression is observable in DM2 muscles compared to controls and this increase is present in DM2-PDM and DM2-PROMM phenotypes but not in DM2-PS. However the CUGBP1 levels in DM2-PDM and DM2-PROMM muscles appear to be lower than those observed in DM1-E2 (fold increase 1.3 *vs* 1.6 compared to controls) (Fig. 3C). Nevertheless, it has been reported that the increase of CUGBP1 steady state protein level in DM1 cultured cells or animal models is related to protein hyperphosphorylation [51]. Several kinases phosphorylate CUGBP1 at different residues and multiple functions of the protein are regulated by phosphorylation at distinct sites. While the specific sites of phosphorylation by PKC have not yet been identified, it has been demonstrated that Akt phosphorylates CUGBP1 at serine-28 (S28) and cyclin D3/cdk4 at serine 302 (S-302) [39], [51], [52]. Since it has been reported that activation of Akt pathway increases CUGBP1 phosphorylation at S-28 in DM1 myoblasts and skeletal muscle, we tested if the increase of CUGBP1 expression observed in our DM cohort is related to an increase of CUGBP1 phosphorylation at S-28. Phosphorylation at S-28 controls nucleus-cytoplasm distribution of CUGBP1, thus it appears that an increase of the expression of CUGBP1-p-S28 isoform may affects CUGBP1 homeostasis since CUGBP1 regulates splicing in the nucleus and stability and translation of mRNA in the cytoplasm, Among all the DM muscles analysed, an increase of CUGBP1-p-S28 was observed only in DM1-E2 which also showed the higher level of CUGBP1 expression. In all other groups, CUGBP1-p-S28 levels are similar to those observed in controls (Fig. 3C). We additionally analysed DM muscle samples through 2D-GE in order to evaluate the CUGBP1 phosphorylation pattern and thus investigate in human biopsies the reported striking protein shift toward a more acidic position previously described in cell system. We analysed CUGBP1 phosphorylation pattern in 12 DM muscle samples confirming an overexpression of CUGBP1 protein only in DM1-E2 respect to control and DM2 biopsies (Fig 3D). In particular, CUGBP1 showed a typical 3 spots pattern in the majority of the sample tested, whereas an additional more acid spot appears only in DM1-E2 sample (Fig. 3D). The appearance of this left spot confirms an increase of the expression of CUGBP1-p form in DM1-E2 patients. Moreover, this additional spot suggests the presence of a more phosphorylated CUGBP1 isoform in DM1-E2, however increased abundance of this isoform should be justified by the overexpression of CUGBP1 in DM1-E2. In order to understand whether CUGBP1 phosphorylation pattern is also altered in DM2 muscles we have compared similar signals of 2D patterns among controls, DM1-E2 and DM2-PROMM to evaluate abundance of different phosphorylation isoforms. As shown in Figure 3E, the CUGBP1 phosphorylation pattern does not show significant alteration in protein spot relative abundance and it does not highlight a hyperphosphorylation of the protein suggesting a similar phosphorylation pattern among the DM phenotype investigated.

*CUGBP1* transcript level was higher in both DM1 and DM2 compared to controls however differences were not statistically significant (Fig 3F).

### ZNF9/CNBP expression is reduced in DM2 muscle biopsies

**Figure 4:**
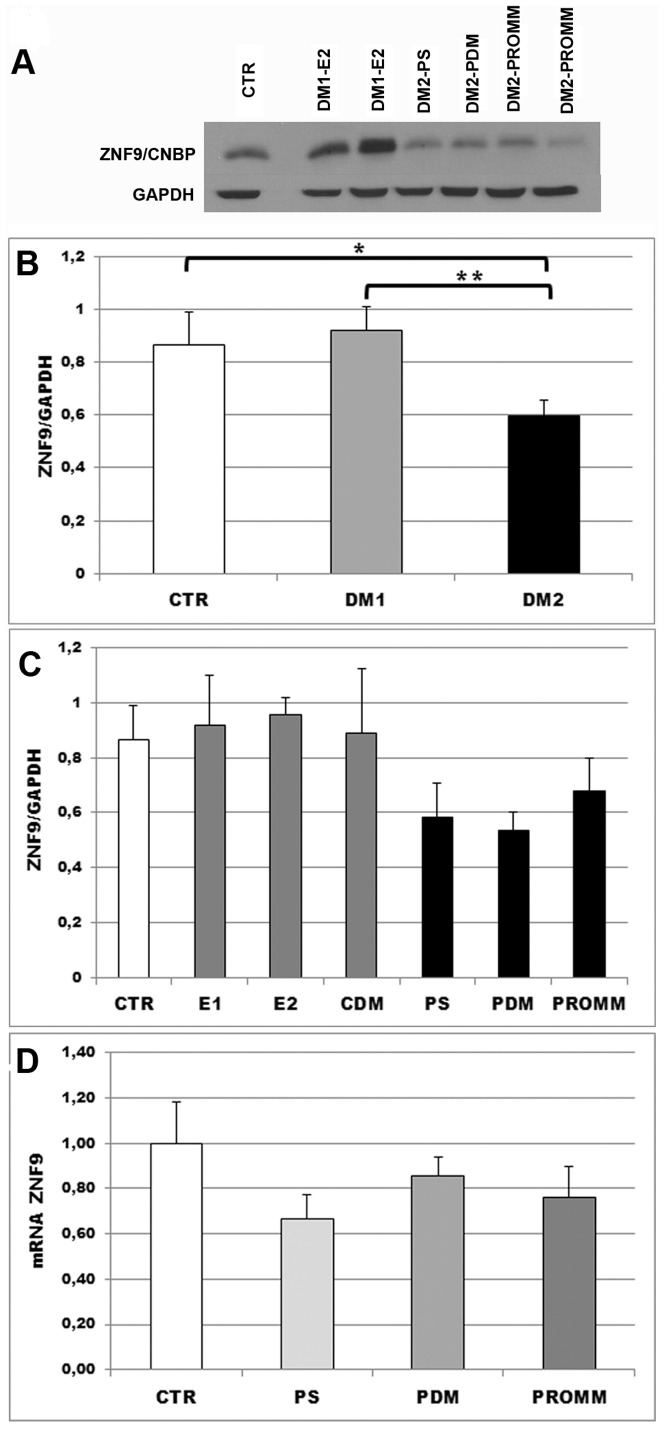
ZNF9/CNBP expression in biceps brachii muscle samples from healthy, DM1 and DM2 patients. **A-C.** ZNF9/CNBP protein expression determined by western blot analysis. **A.** Representative western blot analysis of ZNF9/CNBP protein expression in healthy, DM1 and DM2 patients. Density of the bands has been normalized with GAPDH expression used as internal control. **B.** Histograms represents mean values of ZNF9/CNBP protein expression analysed by densitometry in DM1 (n = 11) and DM2 (n = 14) patients compared to controls (n = 8). ZNF9/CNBP levels are significantly lower in DM2 muscles as compared to DM1 and control muscles. *p<0.05; **p<0.01. **C.** ZNF9/CNBP expression has been also evaluated in DM1 and DM2 phenotypic subgroups (DM1-E1 (n = 3), DM1-E2 (n = 5), DM1-CDM (n = 3), DM2-PS (n = 4), DM2-PDM (n = 5) and DM2-PROMM (n = 5)) compared to controls (CTR; n = 8). The expressions levels of the protein are similar in the three DM2 subphenotypes considered. **D.** ZNF9/CNBP mRNA expression in biceps brachii muscle samples from DM2 patients (DM2-PS (n = 4), DM2-PDM (n = 5) and DM2-PROMM (n = 5)) and controls (CTR, n = 3). mRNA levels are similar in DM2 subgroups. Bars represent SEM.

We have analysed the expression of ZNF9/CNBP at protein and mRNA levels to verify if there is a relationship between their expression and the DM2 clinical severity. ZNF9/CNBP protein levels in examined DM2 muscles are significantly reduced compared with DM1 and control samples whereas the protein level is similar among DM2 subphenotypes (Fig. 4 A-C). Also *ZNF9/CNBP* mRNA expression appears to be lower in DM2 muscle biopsies than in control biopsies (p<0,05; data not shown) and, in agreement with data on protein expression, mRNA levels are similar in the DM2 subtypes considered (Fig. 4D).

### IR, CLCN1, SERCA1, MBNL1 and CAPZB alternative splicing alterations are related to clinical severity of DM subphenotypes

**Figure 5:**
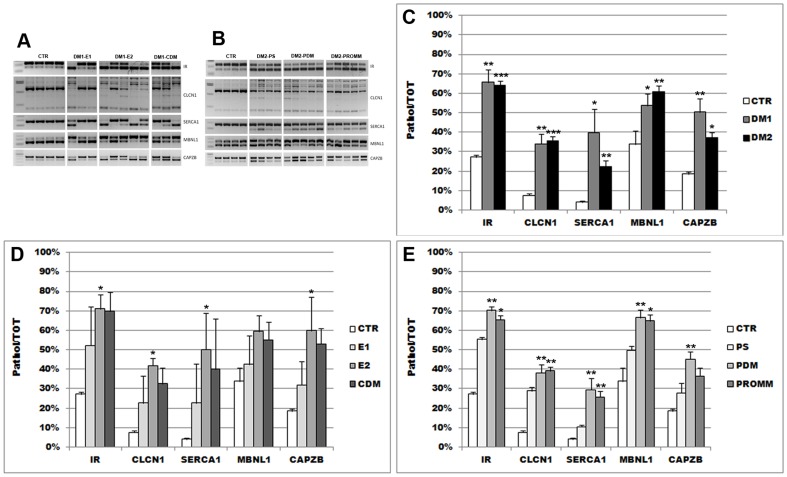
Analysis of alternative splicing of the *IR, CLCN1, SERCA1, MBNL1* and *CAPZB* genes. **A, B.** Splicing products obtained by RT-PCR amplification of RNA isolated from biceps brachii muscle samples from DM1 (A), DM2 (B) and control patients. **C-E**. Densitometric analysis measuring the fraction of aberrant gene isoforms in DM muscles (**C**), and in DM1 (**D**; DM1-E1 (n = 3), DM1-E2 (n = 5), DM1-CDM (n = 3)) and DM2 (E; DM2-PS (n = 4), DM2-PDM (n = 5), DM2-PROMM (n = 5)) phenotypic subgroups compared to controls (CTR). Bars represent SEM; *p<0.05; **p<0.01; ***p<0.001. Alternative splicing of all five genes analysed appear to be altered in DM as compared to non-DM muscles.

In this work we have analysed splicing isoforms of *IR*, *CLCN1*, *SERCA1, MBNL1*and *CAPBZ* genes in muscle biopsy from DM patients to understand if a relationship may exist between the degree of splicing alteration and the phenotype severity. Exons inclusion for all these genes is developmentally regulated and dependent on MBNL1 (*SERCA1* and *MBNL1* genes) [53], [54], CUGBP1 (*CAPZB* gene) [53] or both MBNL1/CUGBP1 proteins (*CLCN1* and *IR* genes) [55]–[57]. We have identified similar defects in *IR*, *CLCN1*, *SERCA1, MBNL1* and *CAPZB* splicing in DM1 and DM2 where the frequency of abnormal isoforms are significantly increased as compared to controls (Fig. 5A-C). The mean percentage of IR-A isoform (*IR*–*A*/*IR*–*A*+*IR*–*B* ratio) in DM1 and in DM2 was 64% and 65% respectively, whereas in controls was 27%. This could explain the insulin resistance in both forms of the disorder. The expression pattern of the *CLCN1* gene has been analyzed across exon 7a, which is abnormally included in DM muscles. In DM1 and DM2 in fact we found 30% and 36% rate of exon 7a inclusion compared to 7% in the control group. *SERCA1* gene is developmentally regulated and SERCA1b isoform, not including exon 22, is characteristic of dystrophic muscle and myotubes [46]. Accordingly to this observation, our DM1 and DM2 samples showed higher level of SERCA1b isoforms than the controls (median percentage of 36% and 22% *vs.* 4% in controls). Similarly, RT-PCR analysis of *MBNL1* splicing pattern across exon 7 region indicated that the ratio of MBNL1 exon 7 inclusion on total *MBNL1* (*MBNLex7*/*MBNLex7*+*MBNL*1Δ7) is 52% in DM1, 59% DM2 and 33% in control samples. On the basis of the observed CUGBP1 increased protein levels in DM1 muscle, we also analyzed the expression of the *CAPZB* gene, which encodes for the F actin capping protein beta subunit. *CAPZB* splicing is dependent only on CUGBP1 and is misregulated in DM1 patients [48], [54]. RT-PCR analysis showed that the ratio of fetal *CAPZB* exon 8-excluding isoform on total *CAPZB* transcripts (*CAPZBΔ8/CAPZBEx8+CAPZBΔ8*) is 48% in DM1, 37% DM2 and 18% in control groups. Interestingly, the DM1-E2 was the category with the highest levels of *CAPZB* Ex8-exclusion transcripts (59%). When considering single phenotypes, DM1-E1 muscles show a lower frequency of abnormal isoforms than those observed in DM1-CDM and DM1-E2 (Fig. 5D). Also in DM2 group, the degree of expression of pathological isoforms in the paucisymptomatic phenotype appears to be lower than those observed in DM2-PDM and DM2–PROMM (Fig. 5E).

It has been shown that the increase of CUGBP1 contributes to the *IR* and C*LCN1* splicing alteration and is the only factor regulating exon inclusion of the *CAPZB* gene [53], [58]. We have found a significant correlation between CUGBP1 protein expression in DM muscles and the frequency of *IR, CLCN1* and *CAPZB* pathological isoforms. A significant correlation is also evident in DM1 cohort but not in DM2 cohort. The increase of CUGBP1 expression significantly correlate with the splicing alterations observed for *SERCA1* and *MBNL1* although CUGBP1 does not seem to be directly involved in splicing misregulation of these genes.

No correlation has been found between the age of patients and the degree of splicing deregulation of the genes examined both in DM1 and DM2 except for *IR* gene in DM2 cohort (p<0,01; data not shown).

Since an elevated concentration of intracellular Ca^2+^ has been suggested to be a possible cause of muscle degeneration [59], we have analysed if there is a correlation between observed histopathological alterations in DM muscles and the expression of pathological isoform of *SERCA1* which is one of the main regulators of intracellular Ca^2+^ homeostasis in skeletal muscle cells. We have found a significant correlation between *SERCA1* splicing alteration and the atrophy factor in DM1 but not in DM2 muscle. A significant correlation between *SERCA1* splicing alteration and hypertrophy factor has been found in DM2.

### CLCN1 mRNA expression levels are similar in DM2 subgroups

**Figure 6:**
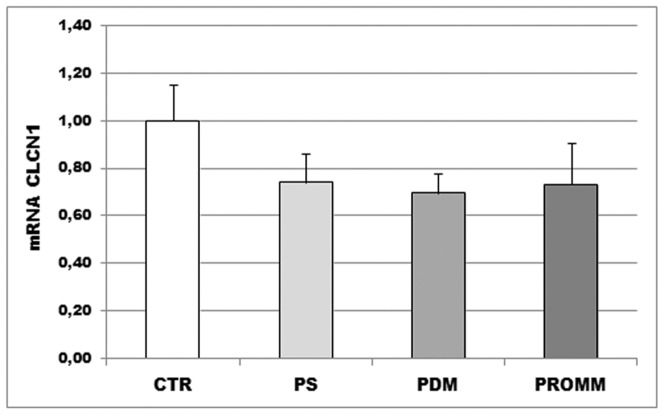
Results of QRT-PCR experiments to quantify the expression level of the *CLCN1* mRNA in biceps brachii muscle samples from DM2 patients (DM2-PS (n = 4), DM2-PDM (n = 5) and DM2-PROMM (n = 5)) and controls. Each experiment has been performed in triplicate and the relative amount of the *CLCN1* transcripts has been determined using the β_2_-microglobulin as endogenous control gene. Bars represent standard deviation.

*CLCN1* mRNA expression levels in DM2 muscle biopsies is reduced as compared to control biopsies (p<0,01, data not shown) and the mRNA levels appear to be significantly lower in DM2-PDM than in controls (Fig. 6). Statistical analysis does not reveal differences in *CLCN1* mRNA expression levels between DM2 phenotypic subgroups considered in this work (Fig. 6). This feature is confirmed also by splicing analysis with densitometry software where DM2-PS, DM2-PDM and DM2-PROMM present about the same level of pathological isoforms.

## Discussion

Myotonic dystrophies are autosomal dominant diseases which share many phenotypic features, however these two disorders also present a number of very dissimilar features making them clearly separate diseases. It is important to underline that DM1 and DM2 phenotypes present a wide clinical spectrum that includes different clinical subphenotypes indicating that molecular pathomechanism of DM is more complex than that actually suggested. The results of our study carried out on skeletal muscle from different DM1 and DM2 subphenotypes seem indicate that the splicing and muscle pathological alterations observed are related to the clinical phenotype. Muscle histopathology of most of DM1 and DM2 patients examined in this work showed the characteristic myopathic features of these diseases. Moreover, in DM2 muscles the mainly affected fiber type is type 2 fibers as previously reported by other authors [29], [60], [61]. However this is true for DM1 and DM2 patient groups showing the more severe multisystemic phenotypes but not for the groups of paucisymptomatic patients, i.e. DM1-E1 and DM2-PS, who present none or minimal muscle histopathological alterations. Moreover as expected, alteration of alternative splicing of *IR*, *CLCN1*, *MBNL1, SERCA1* and *CAPZB* genes is evident in both DM1 and DM2 muscle biopsies despite the clinical phenotype. However it appears that DM1-E1 and DM2-PS patients, who show the less severe clinical and muscle histopathological phenotype, also present a milder spliceopathy profile than those observed in the other DM patients analyzed. It should be noted that the degrees of *CLCN1* splicing misregulation in DM2-PDM is similar to that observed in DM2-PROMM despite myotonia in DM2-PDM is not evident at clinical level. Also, the *CLCN1* mRNA expression levels appear to be similar in DM2-PDM and DM2-PROMM. However it is possible that symptomatic myotonia is not detectable in DM2-PDM patients due to the high degree of atrophy factor observed in skeletal muscle. It is well known that in DM the clinical myotonia is not present at elevated degree of dystrophy or atrophy. Since an elevated concentration of intracellular Ca^2+^ has been suggested to be a possible cause of muscle degeneration [59], we have analysed if there is a correlation between histopathological alterations observed in DM muscles and the expression of pathological isoform of *SERCA1* which is one of the main regulators of intracellular Ca^2+^ homeostasis in skeletal muscle cells. Both in DM1 and DM2 we have found a positive correlation between the muscle alterations and the *SERCA1* splicing alteration thus strengthen the hypothesis that aberrant splicing of this transcript might contribute to severe histopathological alterations in DM patients.

To better define the molecular pathways which may be involved in disease-specific manifestations, we have analysed the role of CUGBP1 particularly intriguing in DM2 since contradictory results have been reported [34]–[36]. While it is clear that MBNL1 is depleted from nucleoplasm through recruitment into ribonuclear inclusions both in DM1 and DM2 even when clinical symptoms and muscle alterations are very mild [35], [62]–[65], CUGBP1 overexpression has been clearly demonstrated in DM1 but not in DM2 muscle biopsies. Our western blotting analysis of CUGBP1 protein expression confirms that CUGBP1 is overexpressed in DM1 muscle biopsies however the increase is evident only in DM1-E2 while CUGBP1 protein levels in DM-E1 and DM1–CDM appear to be similar to those observed in healthy controls. Moreover CUGBP1 overexpression in DM1-E2 biopsies is accompanied by a parallel increase of the amount of phosphorylated isoform. These data correlate with the splicing analysis of the *CAPZB* gene which is regulated specifically by the CUGBP1 protein.

Except for E1-CDM, our data are in line with those reported by Timchenko et al. [66] on DM1 muscle biopsies. In this work, CUGBP1 appear to be overexpressed in CDM and E2 patients but not in E1 patients. However, only one CDM was examined and no mention was made about the age of the patient.

It has been suggested that in DM1 CUGBP1 may be responsible for muscle wasting since the transgenic mice with skeletal muscle-specific expression of CUGBP1 reproduces the dystrophic muscle histology characteristic of DM1 [67] while MBNL1 knockout mice do not exhibit severe muscle wasting suggesting that MBNL1 depletion alone is not able to reproduce this disease feature [68]. In our work we have found a clear correlation between CUGBP1 expression and the atrophy factors found in DM1 muscles. However when considering the different DM1 clinical phenotypes, DM1-E2 and DM1-CDM show the higher values of atrophy factor and the most severe muscle histopathological alterations nevertheless CUGBP1 is overexpressed only in DM1-E2 muscles. It should be noted that the extreme muscle weakness observed in the congenital form of DM1 is not caused by degenerative changes but by developmental defects. Analysis of muscles from CDM patients has shown that muscle fibers are immature in foetuses and that the skeletal muscle maturation is impaired in children [69], [70]. However, the analysis of two successive muscle biopsies of CDM patients showed that in time the muscle is able to gain a certain degree of maturity but never becomes normal since it retains discrepancies in fiber size and the degenerative muscle process begins starting from the second decade when the morphological alterations become identical to those described in late onset myotonic dystrophy [71]. Since we have analysed adult-young CDM patients where muscle histopathological alterations might be due more to the developmental defects than to the degenerative process, it is possible that CUGBP1 expression in our DM1-CDM muscles appears to be similar to DM1-E1 more than to DM1-E2.

Contrary to DM1, in DM2 muscle biopsies examined in this work a slight increase of the CUGBP1 protein levels is observed in DM2-PDM and DM2-PROMM but not in DM2-PS However this increase is not related to an increase of protein phosphorylation. In addition our data on DM2 muscle seem suggest that perturbation of CUGBP1 amount are not required to produce histopathological or splicing regulation defects in DM2. We have observed that the greater expansion in DM2 leads to ribonuclear foci greater than in DM1 which can sequester larger amount of MBNL1. Therefore, the depletion of MBNL1 from nucleoplasm appears to be more extensive in DM2 than in DM1 despite DM1 shows a greater severity of the muscle degeneration (unpublished data). Thus, since sequestration of MBNL1 evidently has a central role in splicing misregulation in both types of DM, it appears likely that in DM1 CUGBP1 overexpression might be an additional pathogenic mechanism not shared by DM2.

It is relevant to highlight that we do not find differences in phosphorylation pattern between the DM phenotypes suggesting that CUGBP1 does not result hyperphosphorylated in DM compared to control muscles. The discrepancy observed between our data on CUGBP1 expression/phosphorylation in DM muscles and those reported by other Authors may be accounted for the model used: measurements made in cultured cells or in animal models which have been used to induce DM pathomechanism may be different from results obtained in human muscle *in vivo.* Moreover differences may exist between different muscle types used.

It has been suggested that also the reduction of ZNF9/CNBP expression in DM2 patients may explain some of the phenotypic disparities between both types of DM. It has been shown that reduction of ZNF9/CNBP levels is sufficient to produce multiorgan symptoms resembling those of DM as observed in heterozygous *Znf9* ^−/−^ knockout mice [72]. We have determined that ZNF9/CNBP protein and mRNA levels in muscle biopsies of biceps brachii from DM2 patients are significantly reduced compared with non-DM2 individuals, including patients with DM1. Our findings are consistent with recent reports of reduced ZNF9/CNBP expression in DM2 [36], [39], [40] and these data indicate that ZNF9/CNBP expression might play a role in phenotypic differences between DM1 and DM2. However ZNF9/CNBP protein appears to be equally expressed in the three DM2 phenotypic groups examined in our work, thus ZNF9/CNBP expression levels do not explain the extreme variability of clinical phenotype evident among DM2 patients. Indeed the expression of ZNF9/CNBP protein in DM2-PS is similar to those observed in DM2-PROMM and DM2-PDM despite paucisymptomatic patients show minor muscle histopathological alterations and the frequency of abnormal isoforms of the genes analysed is lower than in symptomatic patients.

This is the first study on a large number of muscle biopsies from DM1 and DM2 patients analysed at histopathological and biomolecular level. Our results indicate that CUGBP1 seems to play a role in classic DM1 more evidently than in DM2 however no definitive conclusions can be drawn due to the high interindividual variability observed in the different parameters analysed in this study. Nevertheless, it appears that the multisystemic disease spectrum and the phenotypic variability of DM pathologies may not be explained only by spliceopathy thus confirming that the molecular pathomechanism of DM is more complex than that actually appreciate.
